# Self-Perceived Poor/Fair Health, Frequent Mental Distress, and Health Insurance Status Among Working-Aged US Adults

**DOI:** 10.5888/pcd15.170523

**Published:** 2018-07-19

**Authors:** Guixiang Zhao, Catherine A. Okoro, Jason Hsia, Machell Town

**Affiliations:** 1 Division of Population Health, National Center for Chronic Disease Prevention and Health Promotion, Centers for Disease Control and Prevention, Atlanta, Georgia

## Abstract

We examined associations of health insurance status with self-perceived poor/fair health and frequent mental distress (FMD) among working-aged US adults from 42 states and the District of Columbia using data from the 2014 Behavioral Risk Factor Surveillance System. After multiple-variable adjustment, compared with adequately insured adults, underinsured and never insured adults were 39% and 59% more likely to report poor/fair health, respectively, and 38% more likely to report FMD. Compared with working-aged adults with employer-based insurance, adults with Medicaid/Medicare or other public insurance coverage were 28% and 13% more likely to report poor/fair health, respectively, and 15% more likely to report FMD. Increasing insurance coverage and reducing cost barriers to care may improve general and mental health.

## Objective

Self-rated health and health-related quality of life (HRQOL) are commonly used measures of overall well-being, physical health conditions, and functioning (1,2) and are key indicators for assessing national health in Healthy People objectives. Socioeconomic status, lifestyle factors, and chronic conditions affect self-rated health and HRQOL (3,4).

Health insurance improves access to and affordability of care, which can be critical to managing chronic conditions; lacking adequate coverage and not being able to afford care can cause mental distress (5,6). We examined associations of health insurance coverage and type of coverage with self-perceived poor/fair health and frequent mental distress (FMD) among working-aged US adults.

## Methods

The Behavioral Risk Factor Surveillance System (BRFSS) is a state-based, landline-telephone and cellular-telephone survey of noninstitutionalized civilian US adults (7). In 2014, 42 states and the District of Columbia collected health care access data through both core and module questions, which were used for this analysis. The BRFSS protocol was approved by the Centers for Disease Control and Prevention institutional review board. The median response rate was 47.0% in 2014.

Survey participants’ self-perceived health was dichotomized into poor/fair and good/very good/excellent. FMD was defined as having 14 or more days of poor mental health (including stress, depression, and problems with emotions) in the past 30 days.

The survey questions and categorization of participants’ insurance status and type of insurance are described elsewhere (8). Health insurance coverage over the past 12 months was categorized as adequately insured, underinsured, and never insured. Type of coverage was categorized as employer-based, self-purchased, Medicaid or Medicare; other public or some other source, and not currently insured.

Study covariates were age, sex, race/ethnicity, education level, marital status, employment status, federal poverty level (FPL), current smoking, leisure-time physical activity, body mass index, and the number of chronic conditions/diseases including diabetes, coronary heart disease, stroke, current asthma, arthritis, cancer, chronic obstructive pulmonary disease, history of depression, kidney disease, and disability.

Participants who responded “don’t know/not sure,” refused to answer, or had missing responses to any study covariates mentioned above were excluded, leaving 201,781 working-aged adults (aged 18–64 years) in the analytic sample. After further excluding participants with missing data on the 2 outcome variables, 201,423 participants remained for the analysis for self-rated poor/fair health and 199,709 participants remained for the analysis for FMD.

We estimated the weighted prevalence for self-perceived poor/fair health and FMD by health insurance coverage and type of coverage. Log-linear regression analyses were conducted to estimate adjusted prevalence ratios with 95% confidence intervals (CIs) while controlling for study covariates. SAS-callable SUDAAN (Research Triangle Institute) was used to account for the complex survey design. Significance was set at *P* < .05.

## Results

Of 201,781 working-aged adults, the mean age was 41 years; 49.0% were women; 69.9% were non-Hispanic white; 13.5% were non-Hispanic black; and 9.8% were Hispanic. Approximately 59.9% had a degree higher than high school; 14.8% lived below the poverty level (ie, household income <100% of FPL); 20.6% were current smokers; 20.6% were physically inactive; 29.8% were obese; and 48.6% had at least 1 chronic condition or disability.

For insurance status, 57.3% were adequately insured, 32.3% underinsured, and 10.4% never insured. Approximately 56.2% had employer-based coverage; 9.5% had self-purchased coverage; 13.8% had Medicaid or Medicare; 5.3% had other public insurance; and 15.2% were not currently insured.

Overall, 14.8% (95% CI, 14.6%–15.1%) of adults reported poor/fair health, and 12.5% (95% CI, 12.2%–12.7%) reported FMD (Table 1). Prevalence differed by sociodemographic characteristics, lifestyle risk factors, and number of chronic conditions and disability.

**Table 1:** Crude Prevalence of Self-Perceived Poor/Fair Health and FMD Among Working-Aged Adults in 42 States and the District of Columbia, by Sociodemographic Characteristics, Health-Related Behaviors, and Chronic Conditions and Disability, Behavioral Risk Factor Surveillance System, 2014

Characteristic	Poor/Fair Health		FMD	
	No.	% (95% CI)	No.	% (95% CI)
**Overall**	201,423	14.8 (14.6–15.1)	199,709	12.5 (12.2–12.7)
**Age, y**				
18–25	17,710	8.1 (7.5–8.8)	17,580	13.1 (12.3–13.9)
26–44	61,137	12.3 (11.9–12.8)	60,708	12.6 (12.1–13.0)
45–64	122,576	19.9 (19.5–20.3)	121,421	12.1 (11.8–12.4)
**Sex**				
Male	89,898	14.2 (13.8–14.6)	89,154	10.2 (9.8–10.6)
Female	111,525	15.6 (15.2–15.9)	110,555	14.8 (14.4–15.2)
**Race/ethnicity**				
Non-Hispanic white	158,790	13.0 (12.8–13.3)	157,563	12.3 (12.0–12.6)
Non-Hispanic black	18,678	19.3 (18.4–20.2)	18,494	13.8 (13.0–14.7)
Hispanic	12,595	22.6 (21.4–23.9)	12,450	12.4 (11.5–13.4)
Other	11,360	13.4 (12.3–14.5)	11,202	11.2 (10.3–12.2)
**Education level**				
<High school graduate	12,210	34.2 (32.9–35.6)	11,943	21.3 (20.1–22.5)
High school graduate/GED	53,224	18.1 (17.6–18.6)	52,602	13.9 (13.4–14.4)
>High school graduate	135,989	9.6 (9.3–9.8)	135,164	10.1 (9.8–10.4)
**Marital status**				
Married	115,424	11.9 (11.6–12.2)	114,687	8.9 (8.6–9.2)
Previously married	39,528	26.3 (25.6–27.1)	39,010	20.1 (19.4–20.8)
Never married/live with a partner	46,471	14.1 (13.5–14.6)	46,012	14.4 (13.9–15.0)
**Employment status**				
Employed	138,721	8.9 (8.6–9.2)	137,869	8.6 (8.4–8.9)
Unemployed	11,858	22.7 (21.5–24.0)	11,713	21.9 (20.6–23.2)
Not in labor force	50,844	28.7 (28.0–29.4)	50,127	20.2 (19.6–20.8)
**Federal poverty level, %**				
<100	23,075	31.0 (30.0–32.0)	22,759	23.6 (22.7–24.6)
100–400	73,013	16.6 (16.1–17.1)	72,367	13.6 (13.1–14.0)
>400	82,601	6.0 (5.7–6.3)	82,259	6.5 (6.2–6.9)
Unknown	22,734	15.8 (15.1–16.6)	22,324	13.1 (12.3–13.8)
**Current smoking**				
Yes	37,329	24.9 (24.1–25.6)	36,834	23.3 (22.6–24.1)
No	164,094	12.2 (12.0–12.5)	162,875	9.6 (9.4–9.9)
**Leisure-time exercise**				
Yes	161,356	10.8 (10.6–11.1)	160,202	10.4 (10.1–10.6)
No	40,067	30.4 (29.6–31.2)	39,507	20.6 (19.9–21.3)
**Body mass index, kg/m^2^**				
<25.0	68,283	10.1 (9.7–10.5)	67,700	11.3 (10.9–11.8)
25.0–29.9	70,543	12.2 (11.8–12.6)	69,973	10.8 (10.3–11.2)
≥30.0	62,597	23.7 (23.1–24.3)	62,036	15.8 (15.3–16.3)
**Number of chronic conditions and disability** a				
0	92,513	4.4 (4.2–4.7)	92,021	4.8 (4.6–5.1)
1	49,088	10.6 (10.1–11.2)	48,688	11.4 (10.9–12.0)
2	27,245	24.5 (23.6–25.5)	26,951	20.4 (19.5–21.3)
≥3	32,577	54.5 (53.6–55.4)	32,049	37.2 (36.3–38.2)

Abbreviations: CI, confidence interval; FMD, frequent mental distress; GED, general education diploma.

a Including diabetes, coronary heart disease, stroke, current asthma, arthritis, cancer, chronic obstructive pulmonary disease, history of depression, kidney disease, and disability. Disability was defined as respondents who were limited in any way in any activities because of physical, mental, or emotional problems, or who had any health problem that required them to use special equipment (eg, cane, wheelchair, special bed, special telephone).

The age-adjusted prevalence of poor/fair health and FMD was significantly higher among underinsured adults (21.8% and 18.8%, respectively) and never insured adults (23.4% and 16.8%, respectively) compared with adequately insured adults (8.9% and 8.5%, respectively) (Table 2). The age-adjusted prevalence of poor/fair health and FMD were lowest among adults with employer-based insurance (7.8% and 8.5%, respectively) or self-purchased coverage (9.3% and 8.8%, respectively) and highest among adults with Medicaid/Medicare (36.5% and 27.2%, respectively).

**Table 2:** Crude and Age-Adjusted Prevalence and APRs for Self-Perceived Poor/Fair Health and FMD Among Adults Aged 18 to 64 Years in 42 States and the District of Columbia, by Insurance Status and Type of Insurance, Behavioral Risk Factor Surveillance System, 2014

Insurance Status	No.	% (95% Confidence Interval		APR^b^ (%95 CI)
		Crude	Age Adjusted^a^	
**Poor/Fair Health**				
**Insurance coverage during the past 12 months**				
Adequately insured	118,446	9.7 (9.4–10.0)	8.9 (8.6–9.2)	1.00
Underinsured	59,921	22.7 (22.2–23.3)	21.8 (21.3–22.4)	1.39 (1.33–1.44)
Never insured	14,794	22.9 (21.8–24.1)	23.4 (22.3–24.6)	1.59 (1.50–1.68)
**Insurance type**				
Employer-based insurance	116,080	8.3 (8.0–8.6)	7.8 (7.5–8.1)	1.00
Self-purchased plan	19,732	9.8 (9.1–10.5)	9.3 (8.6–10.1)	1.00 (0.93–1.08)
Medicaid or Medicare	26,045	39.2 (38.1–40.2)	36.5 (35.5–37.6)	1.28 (1.22–1.36)
Other public insurance	11,107	18.8 (17.6–20.1)	17.3 (16.1–18.7)	1.13 (1.06–1.21)
Not currently insured	21,368	21.4 (20.5–22.4)	22.5 (21.6–23.4)	1.48 (1.39–1.57)
**FMD**				
**Insurance coverage during the past 12 months**				
Adequately insured	117,630	8.3 (8.1–8.7)	8.5 (8.1–8.8)	1.00
Underinsured	59,296	18.8 (18.3–19.4)	18.8 (18.2–19.3)	1.38 (1.31–1.44)
Never insured	14,625	16.7 (15.7–17.8)	16.8 (15.8–17.8)	1.38 (1.29–1.48)
**Insurance type**				
Employer-based insurance	115,424	8.2 (8.0–8.5)	8.5 (8.2–8.9)	1.00
Self-purchased plan	19,606	9.3 (8.6–10.1)	8.8 (8.1–9.6)	0.95 (0.87–1.04)
Medicaid or Medicare	25,568	27.4 (26.4–28.4)	27.2 (26.1–28.2)	1.15 (1.08–1.23)
Other public insurance^c^	10,976	15.6 (14.5–16.8)	15.8 (14.6–17.2)	1.15 (1.05–1.25)
Not currently insured	21,123	16.7 (15.9–17.6)	16.8 (16.0–17.7)	1.24 (1.16–1.33)

Abbreviation: APR, adjusted prevalence ratio; CI, confidence interval; FMD, frequent mental distress.

a Age adjusted (age groups of 18–25 y, 26–44 y, and 45–64 y were used) to the 2000 projected US population.

b Adjusted for age, sex, race/ethnicity, education level, marital status, employment status, federal poverty level, current smoking, leisure-time physical activity, body mass index, and the number of self-reported, physician-diagnosed chronic conditions (including diabetes, coronary heart disease, stroke, current asthma, arthritis, cancer, chronic obstructive pulmonary disease, history of depression, kidney disease, and disability). Disability was defined as respondents who were limited in any way in any activities because of physical, mental, or emotional problems, or who had any health problem that required them to use special equipment, such as a cane, wheelchair, special bed, or special telephone.

c Includes TRICARE (formerly CHAMPUS), Veterans Affairs, or military plan; Alaska Native, Indian Health Service, Tribal Health Services, or some other source.

After multiple-variable adjustment for study covariates, compared with adequately insured adults, underinsured and never insured adults were 39% (*P* < .001) and 59% (*P* < .001), respectively, more likely to report poor/fair health, and 38% (*P* < .001 for both) more likely to report FMD (Table 2). Compared with adults with employer-based insurance, those with Medicaid/Medicare or other public coverage were more likely to report poor/fair health (28% and 13%, respectively, *P* < .001) and FMD (15% for both, *P* < .001).

## Discussion

Self-assessed health status reflects concurrent decrements in health associated with physical functional status and certain chronic illnesses (1), and lower self-rated health predicts increased mortality (2,9). Poor mental health is associated with risky health behaviors and social burden (6,10). Our results from a large population survey demonstrated that self-perceived health and FMD were significantly associated with health insurance status, independent of socioeconomic status; behavioral risk factors; and multiple chronic conditions and disability. Working-aged adults who were underinsured and never insured or who had Medicaid/Medicare or other public insurance were more likely to rate their general health as poor/fair and to report FMD than their counterparts who were adequately insured or had private insurance.

Research indicates that having insurance coverage predicts better self-rated health (3,11,12) and that prevalence of FMD is significantly higher among adults with no health insurance coverage (5,6) or with financial barriers to needed medical care (5). These results are consistent with our findings that the prevalence of poor/fair health and FMD were significantly higher among underinsured and uninsured adults. Moreover, our results further demonstrated that poor/fair health and FMD were significantly higher among adults with Medicaid/Medicare or other public insurance than among those with private coverage, either employer-based or self-purchased. Adults younger than 65 years who have Medicare or Medicaid are likely to have permanent disabilities or certain terminal diseases, or live in poverty; all of these may contribute to a higher prevalence of poor/fair health or FMD.

The strength of our study is that results are based on a large population surveillance system. BRFSS data, however, are self-reported and subject to recall bias. Also, because BRFSS excluded institutionalized adults, prevalence of poor/fair health and FMD may be underestimated. In addition, data were from 42 states and the District of Columbia, which limits generalizability of the study results to the entire US working-aged population.

Although uninsurance rates are declining in the United States (13), the trend in the rates of being underinsured or having private coverage are largely unknown. Continuing efforts to increase health insurance coverage and reduce cost barriers to needed medical care may help the US population achieve optimal overall health and reduce mental distress.
